# Association Between Nursing Education and Risk of Eating Behavior Disorders Among Undergraduate Students

**DOI:** 10.3390/nursrep15120433

**Published:** 2025-12-07

**Authors:** Edith Araceli Cano-Estrada, José Antonio Guerrero-Solano, Raúl Rodríguez-Moreno, Benjamín López-Nolasco, Sheila Adriana Mendoza-Mojica, Dulce Milagros Razo-Blanco-Hernández, Yaneth Citlalli Orbe-Orihuela, Juan Carlos Fernando Sánchez-Velázquez, Erick Ordoñez-Villordo, José Ángel Hernández-Mariano

**Affiliations:** 1 Superior School of Tlahuelilpan, Autonomous University of Hidalgo Sate, Tlahuelilpan 42780, Mexico; 2 Department of Research, Hospital Juarez of Mexico, Mexico City 07760, Mexico; 3 Department of Chronic Infections and Cancer, Center for Research on Infectious Diseases, National Institute of Public Health, Cuernavaca 62100, Mexico; 4 Nursing School, Hospital Juárez de México, Mexico City 06090, Mexico; 5 Faculty of Medicine, Puebla State University of Health, Puebla of Zaragoza 72000, Mexico

**Keywords:** eating behavior disorders, self-esteem, anxiety, nursing students, undergraduate students

## Abstract

**Background/Objectives**: Eating behavior disorders (EBDs) are a public health concern among undergraduate students. Evidence suggests that certain health-related academic environments may be associated with heightened psychological vulnerability. Hence, we aimed to evaluate the association between nursing education and the risk of EBDs and to assess whether self-esteem and anxiety mediate this relationship. **Methods**: A cross-sectional analytical study was conducted between July and August 2023 among 433 undergraduate students from two public universities in Hidalgo, Mexico. The sample included 209 nursing students and 224 peers from non-health-related programs. Self-esteem, anxiety, and EBD risk were assessed using the Rosenberg Self-Esteem Scale, Hamilton Anxiety Rating Scale, and Eating Attitudes Test-26, respectively. Logistic regression and counterfactual mediation analyses were performed, adjusting for age, sex, family income, and year of study. **Results**: Nursing students showed higher odds of low self-esteem (aOR = 1.64; 95% CI: 1.06–2.53), anxiety (aOR = 2.06; 95% CI: 1.25–3.37), and EBDs risk (aOR = 2.37; 95% CI: 1.37–4.09) compared with non-health peers. Mediation analyses revealed significant indirect effects through self-esteem (aOR = 1.20; 95% CI: 1.03–1.38) and anxiety (aOR = 1.14; 95% CI: 1.01–1.29). **Conclusions**: Nursing education was independently associated with a higher risk of EBDs, with statistical mediation analyses indicating that differences in self-esteem and anxiety may help characterize this association. Self-esteem exerted a slightly stronger indirect effect, suggesting that negative self-evaluation may represent a more proximal psychological process rather than a causal determinant.

## 1. Introduction

Eating behavior disorders (EBDs), also known as eating disorders, encompass a group of psychiatric conditions characterized by persistent disturbances in eating habits, body image, and weight regulation that substantially compromise physical health and psychosocial functioning. The primary diagnostic categories include anorexia nervosa, bulimia nervosa, binge-eating disorder, and other specified feeding or eating disorders [1]. These disorders have a multifactorial etiology, arising from the complex interplay between biological vulnerability, psychological traits, and sociocultural pressures that idealize thinness, perfectionism, and self-control [2].

EBDs are associated with considerable morbidity and mortality, as well as frequent comorbidities such as anxiety, depression, and low self-esteem, making them a growing public health concern—particularly among adolescents and young adults. Globally, it is estimated that nearly 9% of the population has experienced some form of eating disorder, with a markedly higher prevalence among young women [3]. Among undergraduate students, prevalence estimates reach up to 20% [4], approximately doubling the global rate, highlighting the heightened vulnerability of this group to the psychological and social factors that shape body image and self-worth.

During this stage, personality traits, emotional regulation, and self-perception become critical determinants of mental health and eating patterns [5]. Within health-related disciplines, nursing students have been identified as a population at high risk for psychological distress, anxiety, and body image issues [6]. Emerging evidence suggests that nursing students may display greater susceptibility to mental health problems compared to their peers in non-health-related fields, possibly due to the emotional demands, professional expectations, and value systems inherent in health education [7]. However, despite this growing recognition, research specifically addressing the incidence of eating disorders in nursing students compared to other academic groups remains scarce. The strong emphasis on self-discipline, professional appearance, and caring roles inherent in nursing education may further heighten self-examination and pressure to maintain certain physical ideals. Therefore, understanding the psychological mechanisms through which this academic environment may influence EBDs is essential to clarifying how emotional and self-perceptual factors translate into maladaptive eating patterns.

Self-esteem and anxiety represent two psychological constructs that may help explain how the educational context of nursing influences EBDs. Anxiety has been extensively linked to disordered eating, both as a precipitating factor and because of emotional dysregulation and perfectionistic tendencies frequently observed in health-related students [8]. Elevated anxiety levels may lead to maladaptive coping strategies such as restrictive dieting, binge eating, or compensatory behaviors aimed at regaining a sense of control [9,10]. In contrast, self-esteem, defined as the individual’s overall evaluation of self-worth [11], has been less frequently examined as a mediator. Yet, it may play a critical role in this pathway [12]. Low self-esteem can heighten sensitivity to body image concerns and social comparison, increasing vulnerability to internalizing unattainable ideals of thinness or professional appearance. Within nursing education, where students are often exposed to normative expectations of self-control, discipline, and physical presentation [13], diminished self-esteem may therefore act as a novel and modifiable mechanism linking psychological stress to EBDs.

Given the multidimensional nature of EBDs, identifying the contextual and psychological factors that increase vulnerability among specific academic populations is critical. Nursing students, as future health professionals, represent a group of particular interest because their educational environment may simultaneously expose them to high emotional demands and reinforce ideals of self-control and body image [14]. However, most available research has focused on general indicators of psychological distress, with limited attention to the mechanisms underlying disordered eating in this population.

Although previous studies have compared mental health outcomes across different academic disciplines, to our knowledge, no prior research has modeled nursing education as the primary exposure while simultaneously examining its association with low self-esteem, anxiety, and disordered eating. Educating on how self-esteem and anxiety mediate the relationship between nursing education and eating behavior disorders could therefore provide novel insights to guide prevention and mental health promotion strategies within university settings. Thus, we aimed to evaluate the association between nursing education and risk of EBDs among undergraduate students and to assess the role of self-esteem and anxiety in this relationship.

## 2. Materials and Methods

### 2.1. Study Design and Settings

We conducted a cross-sectional analytical study between July and August 2023 at two public universities in Hidalgo, Mexico. These institutions were intentionally selected because they are in the same geographic region, providing comparable sociodemographic and cultural contexts. Only one of the universities offers health-related programs (including Medicine, Nursing, Software Engineering, and Business Administration) while the second offers solely non-health-related degrees such as Engineering and Physical Mathematical Sciences. This structure avoided within-university overlap between Nursing and non-health students and enabled a clear comparison between individuals exposed to a health-oriented academic environment and peers studying in unrelated fields under similar socioeconomic conditions. This design choice was consistent with the study objective and helped minimize potential selection bias.

### 2.2. Population and Study Sample

The target population consisted of 1611 undergraduate students: 776 Nursing students from the university offering health-related programs and 835 students from the university offering non-health-related programs. The minimum sample size was calculated using the formula for relative measures of association (i.e., odds ratio) [15], assuming a 19.7% prevalence of EBDs among university students [4], a minimum detectable odds ratio of 1.86, a 95% confidence level, and an 80% statistical power. Based on these parameters, we estimated a final sample size of 434 participants.

To enhance representativeness within each academic context, we employed a stratified proportional sampling strategy, using the university of origin as the stratification factor. The number of participants selected from each institution was proportional to its enrollment, ensuring adequate representation of both the Nursing program and non-health-related programs. Accordingly, 209 participants were selected from the Nursing university and 225 from the non-health-related university. Within each stratum, participants were randomly selected from the list of eligible students who met the inclusion criteria.

We included male and female students aged 18 years or older enrolled in the specified programs; pregnant women were excluded. All questionnaires were completed, and no participants were excluded from the analysis.

### 2.3. Data Collection

General information was collected through a structured questionnaire that included questions on participants’ sociodemographic characteristics (sex, age, marital status, monthly family income, and whether they had children), as well as academic information (undergraduate program and year of study).

To assess self-esteem, we used the Rosenberg Self-Esteem Scale (RSES) [16], previously validated among Mexican university students and demonstrating acceptable reliability (Cronbach’s alpha = 0.79) [17]. The RSES comprises ten statements, five positive and five negative, scored from 1 (strongly agree) to 4 (strongly disagree), with negative items reverse-scored. The total score ranges from 10 to 40, with conventional cutoffs of 10–27 for low self-esteem, 28–32 for moderate self-esteem, and 33–40 for high self-esteem. In this study, the moderate and high categories were combined into a single group, creating a binary variable that distinguished participants with low self-esteem from those with moderate/high self-esteem. This approach was adopted to increase statistical power and ensure more stable estimates.

Anxiety symptoms were measured using the Hamilton Anxiety Rating Scale (HAM-A), an interviewer-administered instrument consisting of 14 items scored from 0 (absent) to 4 (severe), yielding a total score of 0–56. This scale has been previously used among Mexican university students and has shown acceptable reliability (Cronbach’s alpha = 0.73) [18]. Higher scores indicate greater anxiety severity, with commonly used thresholds classifying 14–17 as without anxiety, 18–24 as moderate, and ≥25 as severe anxiety. For analytical purposes, the moderate and severe categories were combined into a single group, which was subsequently recoded into a dichotomous variable indicating the presence or absence of anxiety.

The risk of EBDs was assessed using the Eating Attitudes Test (EAT-26), an instrument that identifies disordered eating patterns and attitudes toward food, body image, and weight [19]. This instrument has been previously validated in the Mexican population, showing acceptable reliability levels (Cronbach’s alpha = 0.90) [20]. The EAT-26 consists of 26 items rated on a six-point Likert scale ranging from 0 (never) to 6 (always), with item 25 reverse-scored. Individual item scores are summed to obtain a total score ranging from 0 to 78, with higher values reflecting a greater risk of disordered EBDs. The scale evaluates three conceptual dimensions: dieting, which assesses restrictive EBDs and preoccupation with thinness; bulimia and food preoccupation, which measures binge-eating tendencies and obsessive thoughts about food; and oral control, which reflects self-control over food intake and perceived social pressure to gain weight. For analytical purposes, a total score of 20 or higher was classified as at risk for EBDs, whereas scores below 20 were classified as no risk.

### 2.4. Statistical Analysis

The study variables were described using frequencies and percentages. Comparisons of participants’ general characteristics by risk of EBDs were performed using the Pearson chi-square test.

To assess the association between being a nursing student and the risk of EBDs, we applied logistic regression models to estimate the odds of presenting EBDs among nursing students, using students from non-health-related programs as the reference category. The results were expressed as odds ratios (OR) with their corresponding 95% confidence intervals (CIs).

Given theoretical and empirical evidence suggesting that anxiety and self-esteem may participate in the pathway linking academic field with the risk of EBDs, we conducted mediation analyses within the counterfactual framework. Two independent models were estimated using the mediate command in Stata software, version 19.5 (StataCorp, College Station, TX, USA), each evaluating one psychological variable as a potential mediator.

We first estimated the total association between being a nursing student and the risk of EBDs using logistic regression. For each mediator, we then fitted a logistic model predicting the mediator and another model predicting EBDs, including both the exposure and the mediator. This approach allowed us to decompose the total effect into natural direct and indirect effects. Confidence intervals for the direct, indirect, and total effects were obtained via nonparametric bootstrapping with 1000 replications. We reported the total, direct, and indirect effects as odds ratios with 95% confidence intervals, along with the proportion of the total effect explained by the indirect pathway, to facilitate interpretation of each mediator’s relative contribution.

Although the study is cross-sectional, the analytical strategy relies on the conceptual temporal ordering in which the exposure (academic field) precedes the development of psychological states such as self-esteem and anxiety. This assumption supports the plausibility of estimating indirect effects within the counterfactual mediation framework.

All models were adjusted for confounding factors. The selection of confounders for inclusion in the models was based on directed acyclic graphs (DAGs) [21,22,23]. Age, sex, and family monthly income were identified as the minimal sufficient adjustment set for the association between social media addiction and depression (Figure S1).

Variables not selected by the directed acyclic graphs (DAGs) were further evaluated as potential confounders using the change-in-estimate criterion, whereby a variable was considered a confounder if its inclusion in the model produced a meaningful change (≥10%) in the odds ratio for the association between exposure and outcome. The variables religion, marital status, having children, and year of study were assessed using this approach. Among them, only the year of study produced a change greater than 10% in the crude estimate and was therefore retained in the final models. All analyses were performed using STATA software, version 19.5 (StataCorp, College Station, TX, USA).

## 3. Results

Most participants were between 18 and 20 years old. Women predominated (70.4%), and the majority were single (92.8%). Regarding monthly family income, 63.7% reported earning less than US$543.20. Most students were in their third or fourth year of studies. In terms of psychological well-being, 51.3% showed high self-esteem, 73.0% reported anxiety, and a similar proportion presented a risk of developing EBDs (Table 1).

Of the 433 participants, 48.0% were nursing students. When comparing the characteristics of non-healthcare and nursing students, women and individuals from families with lower monthly income were more frequently represented among the latter. Nursing students also showed a higher proportion of low self-esteem, as well as a greater prevalence of anxiety and risk of EBDs compared with their non-healthcare counterparts (Table 1).

On the other hand, when comparing self-esteem and anxiety according to EBD risk status, students with low self-esteem showed a markedly higher prevalence of risk (73.1%) than those with high self-esteem (26.9%; *p* = 0.001). Similarly, anxiety was more common among students at risk (86.0%) compared with those without risk (69.4%; *p* = 0.001; Table 2).

After controlling for potential confounding factors, nursing students showed higher odds of low self-esteem (adjusted odds ratio [aOR] = 1.64; 95% confidence interval [CI]: 1.06–2.53) and anxiety (aOR = 2.06; 95% CI: 1.25–3.37). Consistent patterns were observed in the unadjusted models (Table 3).

Nursing students had higher odds of being at risk for EBDs than their non-health-related peers (aOR = 2.82; 95% CI: 1.73–4.58). Although the association’s magnitude slightly attenuated after adjustment for confounders (aOR = 2.37; 95% CI: 1.37–4.09), it remained statistically significant (Table 4).

**Figure 1:**
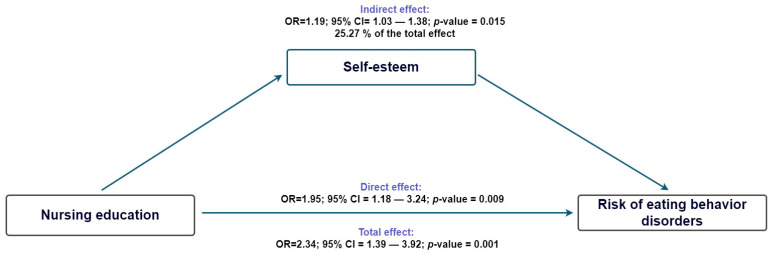
Counterfactual mediation model of nursing education, self-esteem, and Risk of Eating Behavior Disorders.

**Figure 2:**
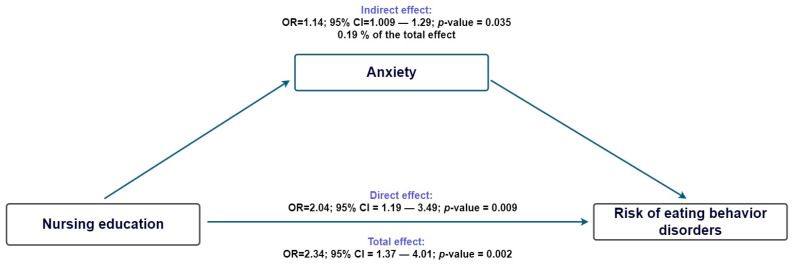
Counterfactual mediation model of nursing education, anxiety, and Risk of Eating Behavior Disorders.

Furthermore, building on these findings, the mediation analysis revealed that self-esteem partially explained the relationship, accounting for 25.2% of the total effect (indirect effect: aOR = 1.20; 95% CI: 1.03–1.38), while the direct effect remained stronger (aOR = 1.95; 95% CI: 1.18–3.24; Figure 1). We also evaluated mediation through anxiety and found a statistically significant indirect effect (aOR = 1.14; 95% CI: 1.01–1.29), which accounted for 19.1% of the total effect. However, the direct effect was greater (aOR = 2.04; 95% CI: 1.19–3.49; Figure 2).

## 4. Discussion

Our findings suggest that nursing students had a higher likelihood of exhibiting risk for EBDs, even after adjusting for sex and other relevant covariates. These results highlight the importance of exploring how the academic environment and psychological distress interact to influence health-related behaviors in this population.

In this study, 48% of nursing students reported low self-esteem, a proportion higher than that described in previous studies conducted in Mexico and other Latin American countries. Among Mexican nursing students, rates of approximately 23.8% have been reported [24], while similar figures (28.9%) have been observed among Peruvian students [25], while similar figures (28.9%) have been observed among Peruvian student. Compared with studies from other regions, the proportion in our sample is also higher; for instance, rates of 23.8% [26] and 27.2% [27] have been reported among Turkish and Saudi nursing students, respectively.

Several contextual factors could help explain these differences, including gender norms, academic workload, institutional stressors, and social expectations that may vary between settings. Methodological factors (e.g., variations in measurement tools, differences in sensitivity and specificity, sample characteristics, and study design) could also contribute to heterogeneity across studies. Additionally, the notably high prevalence of anxiety in our sample may reflect broader stressors faced by Mexican nursing students, including academic pressure, clinical demands, and the psychological impact of training in health-related environments. Nevertheless, both low self-esteem and anxiety remain key psychological constructs affecting undergraduate students worldwide, regardless of cultural or educational context.

Regarding anxiety, the prevalence observed in our study was 81.3%, considerably higher than the rates reported in previous research. A recent umbrella review synthesizing 25 meta-analyses on nursing students reported a pooled global prevalence of 29% (95% CI = 17–40%), with individual studies ranging from 12% to 60%, reflecting substantial heterogeneity across populations [28]. Such discrepancies may reflect specific contextual factors in our sample, including intense academic workload, early exposure to patient suffering, and limited institutional mental health support. Methodological aspects may also contribute, as differences in cutoff scores and data-collection timing (i.e., during examination periods) can inflate prevalence estimates.

In contrast, the prevalence of EBDs risk in our sample (30.3%) was slightly above the upper limit reported in the same meta-analysis, which estimated a pooled prevalence of 19% (95% CI = 8.0–30%) and a range of 20–25% across individual studies [28]. Although our estimate falls within the highest end of previously reported values, this finding underscores the heightened vulnerability of nursing students to maladaptive eating behaviors. Academic pressure, emotional distress, and body image concerns, often intensified by clinical training demands, may jointly contribute to this elevated risk and highlight the need for integrated interventions addressing both mental health and eating-related behaviors in nursing education.

Nursing education was independently associated with low self-esteem, anxiety, and a higher risk of EBDs. These consistent associations remained significant after adjusting for sex and other relevant covariates, suggesting that the emotional and cognitive demands of nursing training may contribute to greater psychological vulnerability. Adjusting for sex was particularly important, as previous research has shown that women are more likely to experience anxiety and to engage in emotional or restrictive eating as coping mechanisms for stress, whereas men tend to externalize psychological distress. Considering that most nursing students are women, controlling for sex allowed us to better isolate the influence of nursing education itself rather than gender-related predispositions to mental health problems.

Moreover, the academic overload, exposure to patient suffering, and empathy-related fatigue that characterize nursing education can erode self-esteem and exacerbate anxiety, thereby increasing susceptibility to maladaptive eating behaviors [7,14]. Although, to our knowledge, no previous studies have modeled nursing education as the main exposure while simultaneously examining its association with low self-esteem, anxiety, and EBDs, other evidence supports our findings. A recent meta-analysis of university students reported a global prevalence of anxiety of approximately 39.6%, which increased substantially among nursing students (48.5%) compared with other disciplines [29]. Similarly, a study of Mexican university students found that nursing students had significantly higher odds of depersonalization, one of the core components of burnout, than peers in non-health-related fields [7], while another comparative study reported higher anxiety levels among nursing students than among those enrolled in medical technology programs [30]. Taken together, these findings suggest that nursing students may experience a unique psychological burden that predisposes them to emotional distress and maladaptive coping behaviors.

Given these consistent associations, we subsequently explored whether anxiety and self-esteem acted as mediating mechanisms linking nursing education with the risk of EBDs. The mediation analyses confirmed that both variables contributed partially and independently to this association.

Previous research outside nursing education has consistently shown that self-esteem and anxiety function as key mediators linking stress exposure, social evaluation, emotional Intelligence, and maladaptive coping behaviors [31,32,33,34,35,36,37]. Low self-esteem has been proposed to heighten vulnerability to disordered eating through processes of self-compensation and perceived loss of control. It attempts to restore self-worth through body image and dietary restriction. In contrast, anxiety mediates these associations primarily through emotional dysregulation and activation of the stress-response system [38,39,40]. The literature indicates that anxiety can trigger hyperactivation of the hypothalamic–pituitary–adrenal (HPA) axis, leading to increased cortisol secretion and subsequent alterations in appetite and reward regulation. Psychologically, anxiety fosters avoidance, hypervigilance, and rumination, which may manifest as restrictive or binge-type EBDs that serve as short-term strategies to manage distress. These complementary pathways provide a theoretical framework that supports the dual mediating role observed in our study [41,42,43].

Interestingly, low self-esteem exerted a slightly more substantial indirect effect than anxiety, accounting for about one quarter of the total association between nursing education and risk of EBDs. This finding suggests that negative self-evaluation may be a more proximal determinant of maladaptive eating behaviors than emotional distress itself. In contrast, anxiety explained a smaller, though significant, proportion of the association, indicating that emotional dysregulation also plays an important but secondary role. Taken together, these results highlight the intertwined yet distinct psychological pathways through which the demands of nursing education may influence students’ vulnerability to disordered eating.

The finding that low self-esteem exerted a stronger mediating role than anxiety between nursing education and the risk of EBDs invites more profound reflection on the unique environment of nursing training. The educational context in nursing is characterized by early and direct exposure to patient care, placing students in high-stakes situations where outcomes are closely tied to human health and welfare [13,44]. Unlike fields where practical training involves equipment or non-life-critical contexts, nursing students frequently perform interventions that directly affect human lives [7,14]. This heightened sense of accountability can undermine self-esteem when students perceive errors or feel underprepared, leading to persistent negative self-evaluation. At the same time, the emotional burden and fear of causing harm can trigger anxiety. Still, because anxiety often reflects immediate emotional arousal, its mediating effect may be more transient or situational [45]. In contrast, self-esteem represents a more stable, internalized sense of self-worth and competence [11]; when it is compromised, students may resort to maladaptive coping behaviors, such as disordered eating, to regain control or self-affirmation. The structural characteristics of nursing education (i.e., intense clinical exposure, empathy fatigue, and high-performance demands) References [13,14,44] may therefore explain why self-esteem emerges as a slightly more potent mediator in this context.

Overall, these findings provide novel information on the psychological mechanisms underlying the link between nursing education and emotional and behavioral health outcomes. However, when interpreting these results, it is essential to first consider that their cross-sectional design precludes establishing causal relationships between nursing education, self-esteem, anxiety, and risk for EBDs. Although our study used a cross-sectional design, this approach was appropriate because nursing education, as an exposure, clearly precedes the psychological constructs measured (self-esteem, anxiety, and EBDs, which can only be assessed at a single time point). Counterfactual mediation models can be validly applied in cross-sectional data when the temporal ordering between exposure and mediators is theoretically justified, as in this educational context. Therefore, our analytic framework maintained temporal coherence despite the study’s cross-sectional design.

Second, the data were collected using self-report instruments, which could introduce misclassification errors; however, since validated scales were used in a Mexican population and administered by standardized personnel, it is unlikely that our findings are affected by reporting bias. Third, the study was conducted in only two public universities within a single Mexican state, which may limit the generalizability of the findings to other institutional or cultural contexts. Although these universities were intentionally selected because they share similar sociodemographic characteristics and because only one offers health-related programs, this design inherently limits representativeness. Using one university with Nursing programs and another offering exclusively non-health-related degrees minimized within-university contamination between groups, but it also means the comparison reflects specific institutional environments. Nonetheless, the psychological mechanisms linking anxiety and eating disorders have been consistently documented across diverse settings, supporting the plausibility of our findings even if contextual differences may affect their magnitude.

Additionally, the demographic profile of our sample, characterized by a young age distribution and a predominance of women, reflects the typical composition of undergraduate nursing programs in Mexico and worldwide. Rather than representing bias, this pattern mirrors the actual structure of the academic population under study. Importantly, sex was included in the minimal sufficient adjustment set identified by the DAG-based confounder selection process, and all multivariable models were adjusted accordingly. Therefore, it is unlikely that the observed associations are driven by demographic imbalance. Nonetheless, generalizability should be interpreted as pertaining to similar academic contexts where nursing programs naturally enroll many women.

Finally, while DAG-guided confounding factor selection and bootstrap-based mediation analysis were applied to strengthen the robustness of our findings, residual confounders cannot be completely ruled out. However, substantial unmeasured confounders are unlikely to have affected the primary association between nursing education and EBDs, given the inclusion of key sociodemographic and contextual covariates in the adjusted models. In contrast, some degree of residual confounding may persist in the associations between anxiety and the risk of BDs, and between self-esteem and such disorders. Specifically, our analyses did not consider potential family factors (i.e., family functioning, parental education, family history of mental health problems) or individual factors (i.e., body mass index) that could influence both self-esteem and anxiety, as well as EBDs Therefore, while our analytical approach was designed to minimize bias, the possibility of residual confounding factors (particularly in psychological pathways) should be recognized when interpreting these results.

## 5. Conclusions

Our findings indicate that nursing students were more likely to experience lower self-esteem, higher anxiety, and a greater vulnerability to risk of EBDs compared with peers from non-health-related programs. Both self-esteem and anxiety appeared to partially mediate the relationship between nursing education and EBDs, with the indirect effect of self-esteem being somewhat more substantial. These results do not imply causality but rather indicate that negative self-evaluation could represent a more proximal psychological process associated with maladaptive eating behaviors than emotional distress itself.

From an educational perspective, these results underscore the need to address self-esteem and emotional regulation within nursing curricula. This recommendation does not imply that such competencies are irrelevant in other academic programs; rather, it reflects that nursing students (who, in our adjusted analyses, exhibited higher levels of psychological vulnerability compared with their non-health-related peers) may particularly benefit from targeted support. Interventions that foster psychological resilience, self-compassion, and adaptive coping strategies could help mitigate the impact of academic stressors and professional expectations on students’ mental health. In addition to formal mental health services and early screening for disordered eating and anxiety, implementing structured tutoring or mentorship programs in which faculty provide individualized academic and emotional guidance may strengthen students’ confidence, self-efficacy, and sense of belonging. Future longitudinal, multi-institutional studies are warranted to clarify causal mechanisms and evaluate the effectiveness of preventive interventions in nursing education.

## Figures and Tables

**Table 1:** Sociodemographic, academic, and psychological characteristics: overall and by education program (non-healthcare students vs. nursing students).

Nursing Students						
Features	Total (n = 433)	No (n = 225)	Yes (n = 208)	Chi-Square	Cramer’s V	*p*-Value
	f (%)	f (%)	f (%)			
Age (in years)						
18–20	237 (54.73)	128 (56.9)	109 (52.4)	0.88	0.05	0.064
21–25	170 (39.27)	84 (37.3)	86 (41.3)			
26	26 (6.0)	13 (5.8)	13 (6.3)			
Sex						
Women	305 (70.44)	119 (52.9)	186 (89.4)	69.28	0.40	0.001
Man	128 (29.56)	106 (47.1)	22 (10.6)			
Marital status				0.50	0.03	
With partner	31 (7.16)	18 (92.0)	13 (6.3)			0.480
No partner	402 (92.84)	207 (8.0)	195 (93.7)			
Having children						
No	409 (94.5)	212 (94.2)	197 (94.7)	0.05	0.01	0.824
Yes	24 (5.5)	13 (5.8)	11 (5.3)			
Practicing religion						
No	27 (6.2)	15 (6.7)	12 (5.8)	0.15	0.02	0.700
Yes	406 (93.8)	210 (93.3)	196 (94.2)			
Family monthly income						
<543.2 American dollars	276 (63.74)	117 (52.0)	159 (76.4)	27.94	0.25	0.001
≥543.2 American dollars	157 (36.26)	108 (48.0)	49 (23.6)			
Years in program						
1–2	181 (41.80)	97 (43.1)	84 (40.4)	0.33	0.03	0.566
3–4	252 (58.20)	128 (56.9)	124 (59.6)			
Self-esteem						
High	222 (51.27)	130 (57.8)	92 (44.2)	7.94	0.14	0.005
Low	211 (48.73)	95 (42.2)	116 (55.8)			
Anxiety						
Absent	117 (27.02)	78 (34.7)	39 (18.7)	13.89	0.18	0.001
Present	316 (72.98)	147 (65.3)	169 (81.3)			
Risk for EBDs						
Absent	117 (27.02)	195 (86.7)	145 (69.7)	18.42	0.21	0.001
Present	316 (72.98)	30 (13.3)	63 (30.3)			

Abbreviations: EBDs, Risk of Eating Behavior Disorders.

**Table 2:** Comparison of self-esteem and anxiety status according to eating disorder risk.

Psychological Variables	Total (n = 433)	Risk of Eating Behavior Disorders				
		No (n = 340)	Yes (n = 93)	Chi-Square	Cramer’s V	*p*-Value
	f (%)	f (%)	f (%)			
Self-esteem						
High	222 (51.27)	197 (57.9)	25 (26.9)	28.20	0.26	0.001
Low	211 (48.73)	143 (42.1)	68 (73.1)			
Anxiety						
Absent	117 (27.02)	104 (36.6)	13 (14.0)	10.22	0.15	0.001
Present	316 (72.98)	236 (69.4)	80 (86.0)			

**Table 3:** Crude and Adjusted Odds Ratios for the Association between Nursing Program and Low Self-Esteem and Anxiety.

Nursing Students	Self-Esteem				Anxiety			
	OR (CI 95%)	*p*-Value	OR (CI 95%) ^a^	*p*-Value	OR (CI 95%)	*p*-Value	OR (CI 95%) ^a^	*p*-Value
No	Ref.		Ref.		Ref.		Ref.	
Yes	1.72 (1.17–2.52)	0.005	1.64 (1.06–2.53)	0.023	2.29 (1.47–3.58)	0.001	2.06 (1.25–3.37)	0.004

Abbreviations: ref., reference. ^a^ Model adjusted for age, sex, family monthly income, and years in the education program.

**Table 4:** Crude and Adjusted Models of the Association between Nursing Education and Risk of Eating Behavior Disorders.

Nursing Students	Risk of Eating Behavior Disorders			
	OR (CI 95%)	*p*-Value	OR (CI 95%) ^a^	*p*-Value
No	Ref.		Ref.	
Yes	2.82 (1.73–4.58)	0.001	2.37 (1.37–4.09)	0.002

Abbreviations: ref., reference. ^a^ Model adjusted for age, sex, family monthly income, and years in the education program.

## Data Availability

The original data presented in the study are openly available in Mendeley Data at doi: https://data.mendeley.com/datasets/8f66fjkg5b/1 (accessed on 31 October 2025).

## Supplementary Materials

The following supporting information can be downloaded at: https://www.mdpi.com/article/10.3390/nursrep15120433/s1, Figure S1: Directed acyclic graph of the hypothesized mediation model.

## Public Involvement Statement

No public involvement in any aspect of this research.

## Guidelines and Standards Statement

This manuscript was drafted against the Strengthening the Reporting of Observational Studies in Epidemiology (STROBE) checklist for cohort research.

## Abbreviations

The following abbreviations are used in this manuscript:

EBDs	Eating behavior disorders
aOR	Adjusted odds ratio
OR	Odds Ratio
CI	Confidence Intervale
RSES	Rosenberg Self-Esteem Scale
EAT-26	Eating Attitudes Test
HAM-A	Hamilton Anxiety Rating Scale
