# Supplementary material for: Association Between Nursing Education and Risk of Eating Behavior Disorders Among Undergraduate Students
Source: Nurs Rep. 2025 Dec 7;15(12):433. doi: 10.3390/nursrep15120433 (PMC12735860; doi:10.3390/nursrep15120433)
Supplement: Supplementary file 1 [file nursrep-15-00433-s001.zip › nursrep-3990659-supplementary.pdf]

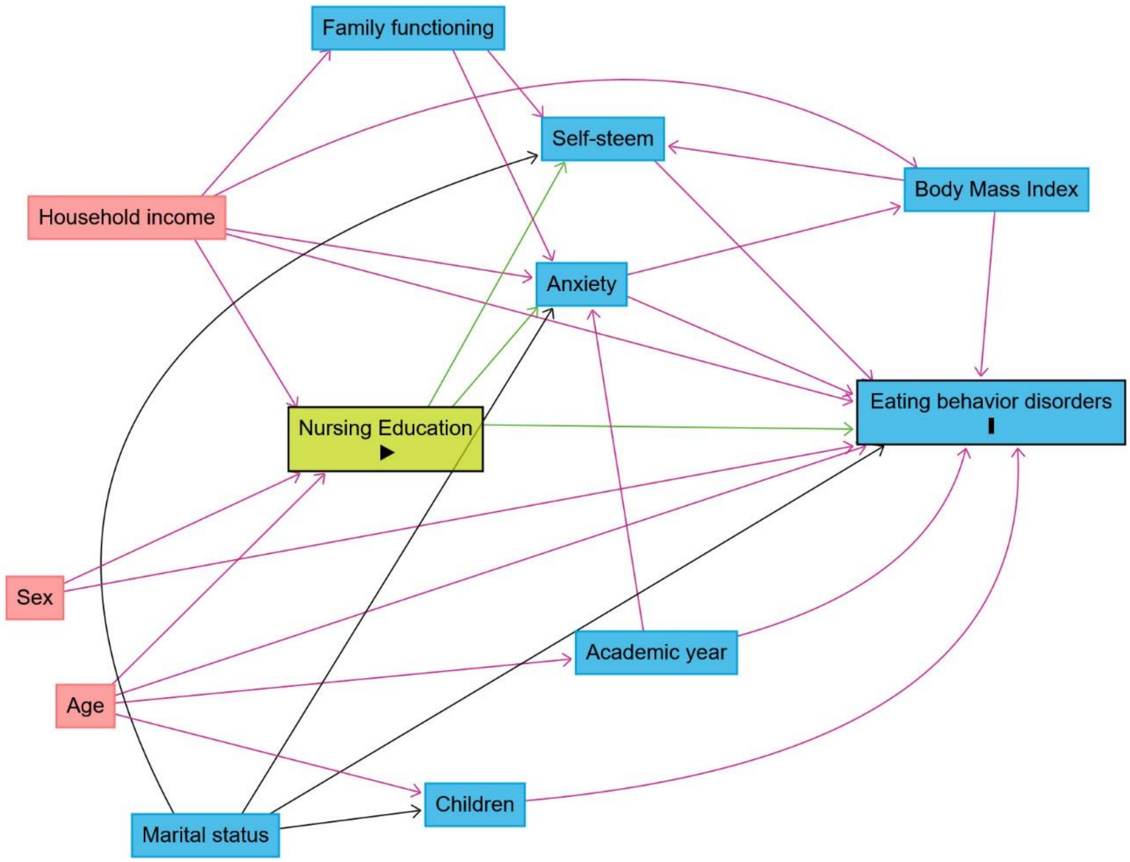

Figure S1. Directed acyclic graph of the hypothesized mediation model. Directed acyclic graph for the hypothesized counterfactual mediation structure in which nursing education influences eating behavior disorders directly and indirectly through self-esteem and anxiety.
